# Remodeling of Aortic Configuration and Abdominal Aortic Branch Perfusion After Endovascular Repair of Acute Type B Aortic Dissection: A Computed Tomographic Angiography Follow-Up

**DOI:** 10.3389/fcvm.2021.752849

**Published:** 2021-10-25

**Authors:** Zihui Yuan, Yiqing Li, Bi Jin, Jian Wang

**Affiliations:** Department of Vascular Surgery, Union Hospital, Tongji Medical College, Huazhong University of Science and Technology, Wuhan, China

**Keywords:** aortic dissection (AD), thoracic endovascular aortic repair, aortic remodeling, true lumen, false lumen, abdominal aorta, computed tomographic angiography

## Abstract

**Background:** Thoracic endovascular aortic repair (TEVAR) for type B aortic dissection (TBAD) induces false lumen (FL) thrombosis, promotes favorable aortic remodeling, and makes an impact on abdominal aortic branch perfusion patterns. However, little is known about the long-term fate of aortic remodeling and abdominal aortic branch perfusion after TEVAR for TBAD and the effect of FL thrombosis status on these changes.

**Materials and methods:** Between January 2014 and May 2021, 59 enrolled patients with acute TBAD were treated with TEVAR and had post-operative or follow-up images. Pre-operative, post-operative, and latest follow-up CT angiography (CTA) data were analyzed for the largest diameter of true lumen (TL), FL, and transaorta and for the FL thrombosis status on the stented thoracic aorta, unstented thoracic aorta, and abdominal aorta. Abdominal aorta perfusion patterns were characterized.

**Results:** The mean follow-up period was 17.1 months. In the stented thoracic aorta, average TL diameters increased, average FL diameters decreased, and average transaortic diameters did not change; 82.6% of the patients had either a stable or shrinking transaortic size and 87% of the patients achieved total FL thrombosis. In the unstented thoracic aorta, average TL diameters increased, transaortic growth and no changes occurred in 39.1 and 45.7% of the patients, respectively, and complete FL thrombosis was present in 50% of the patients. In the abdominal aorta, average FL and transaortic diameters increased, aorta was expanded in 52.2% of the patients, and FL remained patent in 65.2% of the patients. Of the 354 branches, 37 branches (10.5%) exhibited changes in perfusion patterns, 22 branches (6.2%) demonstrated an increased TL perfusion, and 15 branches (4.2%) had an increased FL contribution. Compared with patent or partially thrombosed FL, complete FL thrombosis was accompanied by a bigger decrease in FL diameters, a larger increase in TL diameters, and a higher percentage of abdominal branch TL perfusion.

**Conclusions:** In majority of the patients, TEVAR stabilized the size of the stented thoracic aorta, namely TL expansion and FL obliteration. However, abdominal aortic FL remained patent FL, and it was expanded with the resultant transaortic growth over a long follow-up period. Abdominal aortic branch perfusion patterns remained largely stable after TEVAR. The failure to achieve FL thrombosis negatively affects the remodeling of a contagious abdominal aortic dissection.

## Introduction

Thoracic endovascular aortic repair (TEVAR) with stent graft coverage of a proximal entry tear was firstly reported for an aortic dissection in 1999 (1). Rapidly accumulating data in the last decade have confirmed that TEVAR has been widely used for complicated type B aortic dissection (TBAD) (2, 3). Several clinical trials have reported that TEVAR offers improved all-cause and aorta-specific survival, delayed disease progression, and decreased an associated complication for TBAD compared with pure drug treatment (3) or open repair (4, 5).

Thoracic endovascular aortic repair for TBAD reduces false lumen (FL) blood flow, induces FL thrombosis, and allows endograft-assisted true lumen (TL) expansion by the closure of the primary intimal tear with the placement of a covered stent graft into TL. Favorable aortic remodeling includes TL expansion, FL regression and thrombosis, and overall aortic diameter stabilization. Complete FL thrombosis, mainly in the stent graft-covered thoracic aorta, may invoke positive remodeling, whereas a patent FL correlates with a higher risk of aortic dilation and death (6). Aortic remodeling after TEVAR in acute and chronic TBAD (7) as well as in DeBarkey class IIIa and IIIb dissections (8) has been intensively investigated. We sought to characterize structural changes (aortic remodeling) in the stented thoracic aorta, unstented distal thoracic aorta, and abdominal aorta after the performance of TEVAR for acute TBAD. Of special interest is the effect of TEVAR-induced FL thrombosis on TL, FL, transaortic diameters of the dissected aorta.

Thoracic endovascular aortic repair rechannels blood flow into TL by sealing a proximal entry tear, and depressurizes FL. This hemodynamic alteration raises a concern for the potential impact of proximal TEVAR on the downstream aortic branches, particularly the ones supplied by FL. Little information is available on the impact of TEVAR on abdominal aortic branches when it is used in TBAD. Thus, we investigated abdominal aortic branch perfusion patterns at baseline and their changes after TEVAR.

## Patients and Methods

### Patients

This study was conducted with an approval from the institutional review board of Union Hospital, Tongji Medical College, Huazhong University of Science and Technology, Wuhan, China. As this study was a retrospective review, no informed consent was required. From January 2014 to May 2021, 183 patients with TBAD underwent TEVAR at our department, of which 59 (32.2%) patients who met the following criteria were included in this study: (1) dissection involving the aorta below the celiac artery (CA) level, (2) treatment performed within the acute phase (up to 14 days from symptom onset), (3) partially or completely patent FL at the time of intervention, and (4) available CT scans on admission, before hospital discharge, and/or in a follow-up period. Around 67.8% (124/183) of the patients were excluded for the following reasons: (1) a dissection involving a level above the CA (*n* = 4), (2) the subacute and chronic dissection (?15 days from the initial symptom) (*n* = 7), (3) the absence of FL contrast enhancement (*n* = 7), (4) non-post-operative and/or follow-up CT angiography (CTA) available (*n* = 106). Clinical data were collected from the hospital electronic medical records. An aortic dissection not extending to the abdominal aorta was excluded. Intraluminal hematomas, penetrating ulcers, connective tissue disease (e.g., Marfan syndrome), or traumatic aortic injury were not eligible for this study.

### TEVAR Procedure

The procedure is performed using transfemoral access in an endovascular operating suite equipped with digital subtraction angiography by participating vascular surgeons. Endografts used in this series were oversized by 10% and consisted of Valiant (*n* = 41; Meditronic, Minneapolis, MN, USA), C-TAG (*n* = 3; Gore, Flagstaff, AZ, USA), Hercules (*n* = 8; Microport, Shanghai, China), and Ankura (*n* = 7; Lifetech, Shenzhen, China). Stent graft selection was dependent on the length of aortic coverage, proximal and distal landing zone diameter, single fenestration for the left subclavian artery (LSA), the preference of a patient, and the familiarity of an operating surgeon. The stent graft was first deployed to cover the primary entry tear. Additional stent graft was placed only when the initial graft was insufficient to reestablish flow into TL or was inadequate for the primary entry coverage. When TBAD involves the aortic arch or there was no sufficient proximal landing zone, chimney stent implantation or single fenestration for the LSA, carotid–carotid bypass artery, or carotid-subclavian artery was performed. If TEVAR alone did not resolve end-organ malperfusion, bare-metal stents would be placed at the origin of visceral and renal vessels.

### Definition

Aortic dissections are also classified based on chronicity. Acute dissection refers to <2 weeks from symptom onset. Subacute dissection and chronic dissection refer to 2–6 weeks and more than 6 weeks from symptom onset, respectively. Changes in TL, FL, and transaortic diameters after TEVAR and during follow-up period were defined as growth (>a 3- mm increase), stabilization (≤a 3-mm increase or decrease), shrinkage (>3 mm decrease) at a post-procedural or follow-up time point in comparison with the baseline measurement during a pre-procedural examination.

### Aortic Morphology Analysis

CT angiography was performed at presentation and post-operation, after 6 months, and then yearly thereafter. The pre-TEVAR, the post-TEVAR, and the latest follow-up CTA were reviewed and compared for research analysis among the selected patients. The degree of aortic remodeling with FL thrombosis after TEVAR was evaluated in three separate aortic segments: the stented thoracic aorta, the thoracic aorta distal to the stent, and the abdominal aorta. Measurements, namely, the largest inner diameter of the lumen, the maximum inner diameter of TL and FL, and total blood vessel diameter, were obtained by manual segmentation. The maximum diameter of the aorta was evaluated inner-wall to inner-wall on a cross-sectional image. The maximum diameter of TL and FL was measured perpendicular to the contour of the intimal flap on axial CT scans. FL status was qualitatively assessed on CTA as complete thrombosis (evidence of thrombus without contrast evidence), partially thrombosed (evidence of both contrast and thrombus), or patent (contrast evidence without evidence of thrombus). All measurements were obtained by two experienced radiologists with >3 years in cardiovascular imaging, and final decisions were reached by a consensus.

### Brach Vessel Analysis

Celiac trunk, superior mesenteric artery (SMA), bilateral renal arteries, and bilateral common iliac arteries were analyzed. Each branch perfusion pattern was characterized as being supplied by TL, FL, or both lumens (BL). Changes in branch perfusion were determined by comparing baseline, post-TEVAR, and follow-up CTA images. The branch perfusion pattern was defined as positive when there was an increased TL and a decreased FL contribution to the blood supply. For example, a branch whose perfusion pattern changed from BL to only TL after TEVAR was characterized as positive. Likewise, branches changed from only FL perfusion to both TL and FL were also classified as positive. Conversely, the branch perfusion pattern was defined as negative when FL perfusion increased with the loss of TL perfusion or branches with TL or FL contributions to perfusion occluded after TEVAR.

### Statistical Analysis

Continuous variables were summarized as mean (SD). Categorical variables were expressed as frequency and percentage. Statistical analysis was performed using the SPSS statistical software, version 23.0 (IBM Corp., Armonk, NY, USA). Fisher's exact test for categorical variables and one-way ANOVA or a non-parametric test for continuous variables was used to determine a statistical difference in variables between groups. Differences of *p* < 0.05 were considered as statistically significant.

## Results

### Baseline Characteristics

Table 1 shows the demographic profiles, comorbidities, and baseline performance of patients. A total of 59 patients were included in this study. The mean age of patients was 53.1 ± 11.3 years, and 53 (89.8%) patients were men. Hypertension is present in 50 (84.7%) of the study population. Other complications, such as coronary heart disease, diabetes, chronic obstructive pulmonary disease, and renal insufficiency, were rare.

**Table 1:** Demographic features, comorbidities, and pre-operative presentation of patients with TEVAR.

**Patients**	***n* = 59**
**Baseline characteristics**	
Age (years)	53.1 ± 11.3
Male	53 (89.8%)
**Comorbidities**	
Hypertension	50 (84.7%)
Diabetes	1 (1.6%)
COPD	1 (1.6%)
CAD	5 (8.2%)
Renal insufficiency	2 (3.3%)
**Pre-operative presentation**	
Pain	56 (94.9%)
Visceral malperfusion	1 (1.7%)
Lower extremity malperfusion	1 (1.7%)
Aneurysmal degeneration	1 (1.7%)
Other	3 (5.1%)

*TEVAR, thoracic endovascular aortic repair; COPD, chronic obstructive pulmonary disease; CAD, coronary artery disease*.

Majority of the patients (94.9%, 56/59) were presented with sudden chest and back pain. Two (3.4%) patients had a poor perfusion of organs, including lower limbs (one case), intestines, and kidneys (one case). Three (5.1%) patients had no obvious clinical symptoms, TEVAR was selected after abnormalities were found at a physical examination.

One patient developed a new entry after TEVAR and underwent the second intervention. One patient was readmitted to the hospital for a percutaneous endoleak repair due to the finding of an endoleak 4 months after TEVAR. Among the patients included in this study, there were no deaths due to a dissection within 30 days.

### Aortic Morphology: TL, FL, and Transaortic Diameters

**Figure 1:**
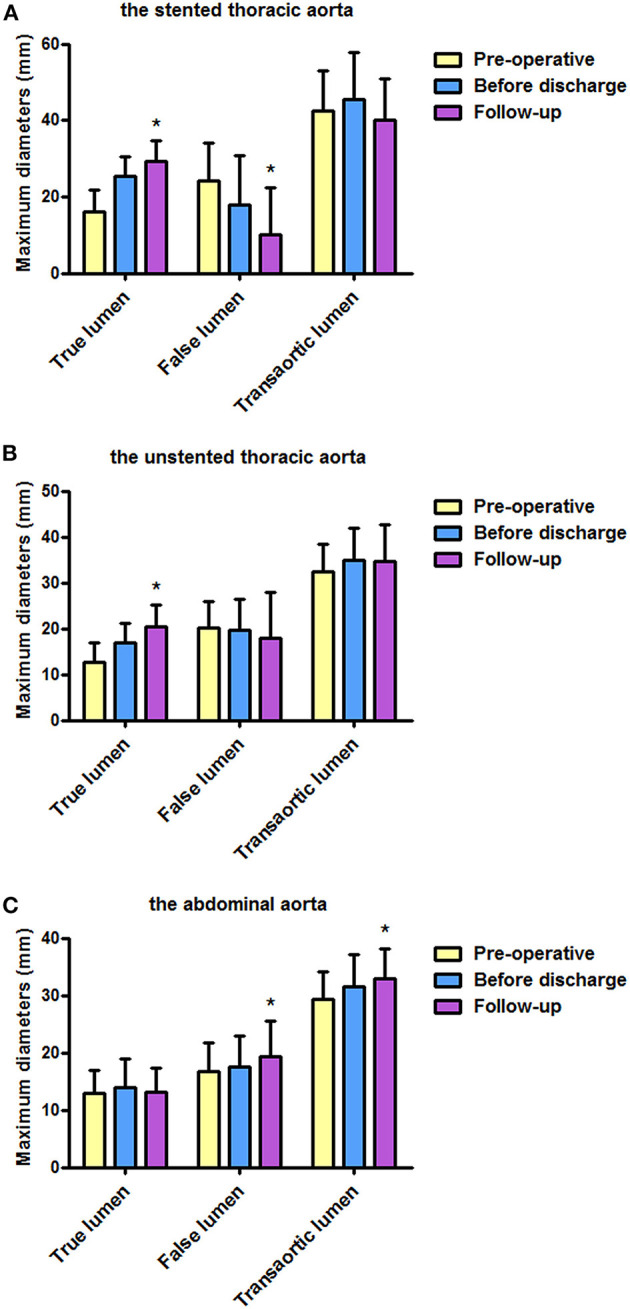
Maximum diameters of FL, TL, and transaorta pre-operatively, before discharge, and during follow-up. **(A)** Average maximum diameters of the stented thoracic aorta were stable, average diameters of the stented thoracic TL increased, and average diameters of the stented thoracic FL decreased. **(B)** Average diameters of the unstented thoracic TL increased. **(C)** An increase in average maximum diameters observed in the abdominal aorta was mainly attributed to FL enlargement. FL, false lumen; TL, true lumen. *P < 0.05 versus pre-operative or before discharge.

Table 2 shows the maximum lumen inner diameters of FL, TL, and transaorta pre-operatively, immediately before discharge and during a most recent follow-up. Maximum lumen inner diameters on the stented thoracic aorta (A), unstented thoracic aorta (B), and abdominal aorta (C) are shown in Figure 1.

**Table 2:** Maximum diameters of the true lumen (TL), false lumen (FL), and total lumen (transaortic) over time in the stented thoracic aorta, unstented thoracic aorta, and abdominal aorta.

**Variables**	**Pre-operative**	**Before discharge**	**Follow-up**	***P-*value**
**The stented thoracic aorta**				
True lumen	16.0 (5.7)	25.5 (5.0)	29.4 (5.4)	<0.01
False lumen	24.3 (9.8)	17.9 (12.9)	10.0 (12.3)	<0.01
Transaortic lumen	42.4 (10.6)	45.4 (12.4)	40.1 (10.8)	0.098
**The unstented thoracic aorta**				
True lumen	12.6 (4.4)	16.8 (4.3)	20.3 (4.8)	<0.01
False lumen	20.2 (5.8)	19.6 (6.8)	17.9 (10.1)	0.574
Transaortic lumen	32.5 (6.0)	34.9 (6.9)	34.7 (8.0)	0.158
**The abdominal aorta**				
True lumen	12.9 (4.1)	14.0 (4.9)	13.1 (4.2)	0.425
False lumen	16.7 (5.0)	17.5 (5.5)	19.4 (6.2)	0.046
Transaortic lumen	29.4 (4.7)	31.5 (5.7)	32.9 (5.3)	0.006

*All data are expressed as means (SD) in mm*.

### The Stented Thoracic Aorta

Average TL diameter increased significantly from 16.0 mm pre-operatively to 25.5 mm before discharge and then continued to increase up to 29.4 mm duringfollow-up (*p* < 0.01). Average FL diameter decreased substantially from 24.3 mm pre-operatively to 17.9 mm before discharge, and it was 10.0 mm during follow-up (*p* < 0.01). Average transaortic diameter increased from 42.4 mm pre-operatively to 45.4 mm before discharge and then reduced to 40.1 mm during follow-up (*p* = 0.098).

### The Unstented Thoracic Aorta

Average TL diameter increased over time as well from 12.6 mm pre-operatively to 16.8 mm and then increased to 20.3 mm during follow-up (*p* < 0.01). Average FL diameter decreased slightly over time but did not reach a statistical significance (*p* = 0.574). Average transaortic diameter increased from 32.5 mm pre-operatively to 34.9 mm before discharge, and then it was relatively stable (34.7 mm) during follow-up (*p* = 0.158).

### The Abdominal Aorta

Average TL diameter did not change over time (*p* = 0.425). Average FL diameter was 16.7 and 17.5 mm pre-operatively and before discharge, respectively, but increased to 19.4 mm during follow-up (*p* = 0.046). Average transaortic diameter slightly raised from 29.4 mm pre-operatively to 31.5 mm before discharge, and it was eventually grown to 32.9 mm during follow-up (*p* = 0.006).

### Aortic Morphology: Changes in TL, FL, and Transaortic Diameters

Changes in maximum TL, FL, and transaortic diameters were further categorized into increase (>3 mm), decrease (<3 mm), and stable within 3 mm, as shown in Table 3.

**Table 3:** Changes in the maximum diameters of the TL, FL, and total lumen (transaortic) in the stented thoracic aorta, unstented thoracic aorta, and abdominal aorta, which are categorized as increase, stable, and decrease.

**Variable**	**Post-operative**	**Follow-up**
**The stented thoracic aorta**		
True lumen		
Increase	34 (87.2%)	44 (95.7%)
Stable	5 (12.8%)	2 (4.3%)
Decrease	0 (0%)	0 (0%)
False lumen		
Increase	2 (5.1%)	4 (8.7%)
Stable	8 (20.5%)	5 (10.9%)
Decrease	29 (74.4%)	37(80.4%)
Transaortic lumen		
Increase	14 (35.9%)	8 (17.4%)
Stable	22 (56.4%)	24 (52.2%)
Decrease	3 (7.7%)	14 (30.4%)
**The unstented thoracic aorta**		
True lumen		
Increase	25 (64.1%)	39 (84.8%)
Stable	14 (35.9%)	6 (13.0%)
Decrease	0 (0.0%)	1 (2.2%)
False lumen		
Increase	6 (15.4%)	10 (21.7%)
Stable	24 (61.5%)	19 (41.3%)
Decrease	9 (23.1%)	17 (37.0%)
Transaortic lumen		
Increase	13 (33.3%)	18 (39.1%)
Stable	25 (64.1%)	21 (45.7%)
Decrease	1 (2.6%)	7 (15.2%)
**The abdominal aorta**		
True lumen		
Increase	7 (17.9%)	8 (17.4%)
Stable	28 (71.8%)	32 (69.6%)
Decrease	4 (10.3%)	6 (13.0%)
False lumen		
Increase	9 (23.1%)	21 (45.7%)
Stable	24 (61.5%)	20 (43.5%)
Decrease	6 (15.4%)	5 (10.9%)
Transaortic lumen		
Increase	11 (28.2%)	24 (52.2%)
Stable	27 (69.2%)	21 (45.7%)
Decrease	1 (2.6%)	1(2.2%)

*All data are expressed as n (%)*.

### The Stented Thoracic Aorta

True lumen diameters were increased in 87.2% of the patients before discharge and in 95.7% of the patients during follow-up. Around 74.4 and 80.4% of the patients had a decreased FL diameter before discharge and during follow-up period, respectively. Consequently, transaortic diameters were stable or decreased for 64.1 and 82.6% of the patients before discharge and during follow-up, respectively.

### The Unstented Thoracic Aorta

True lumen diameters were increased in 64.1% of the patients before discharge and in 84.8% of the patients during follow-up. FL diameters were stable or decreased in 84.6% of the patients before discharge and 78.3% of the patients during follow-up. Therefore, 97.4 and 84.8% of the patients had either stable or increased transaortic diameters before discharge and during follow-up, respectively.

### The Abdominal Aorta

True lumen diameters were stable in 71.8% of the patients before discharge and 79.6% of the patients during follow-up. Around 84.6and 89.2% of the patients had either stable or increased FL diameters before discharge and during follow-up, respectively. Eventually, transaortic diameters were stable for 69.2 and 45.7% of the patients before discharge and during follow-up, respectively. Around 28.2 and 52.2% of the patients had increased transaortic diameters before discharge and during follow-up, respectively.

### Aortic Morphology: FL Status

**Figure 2:**
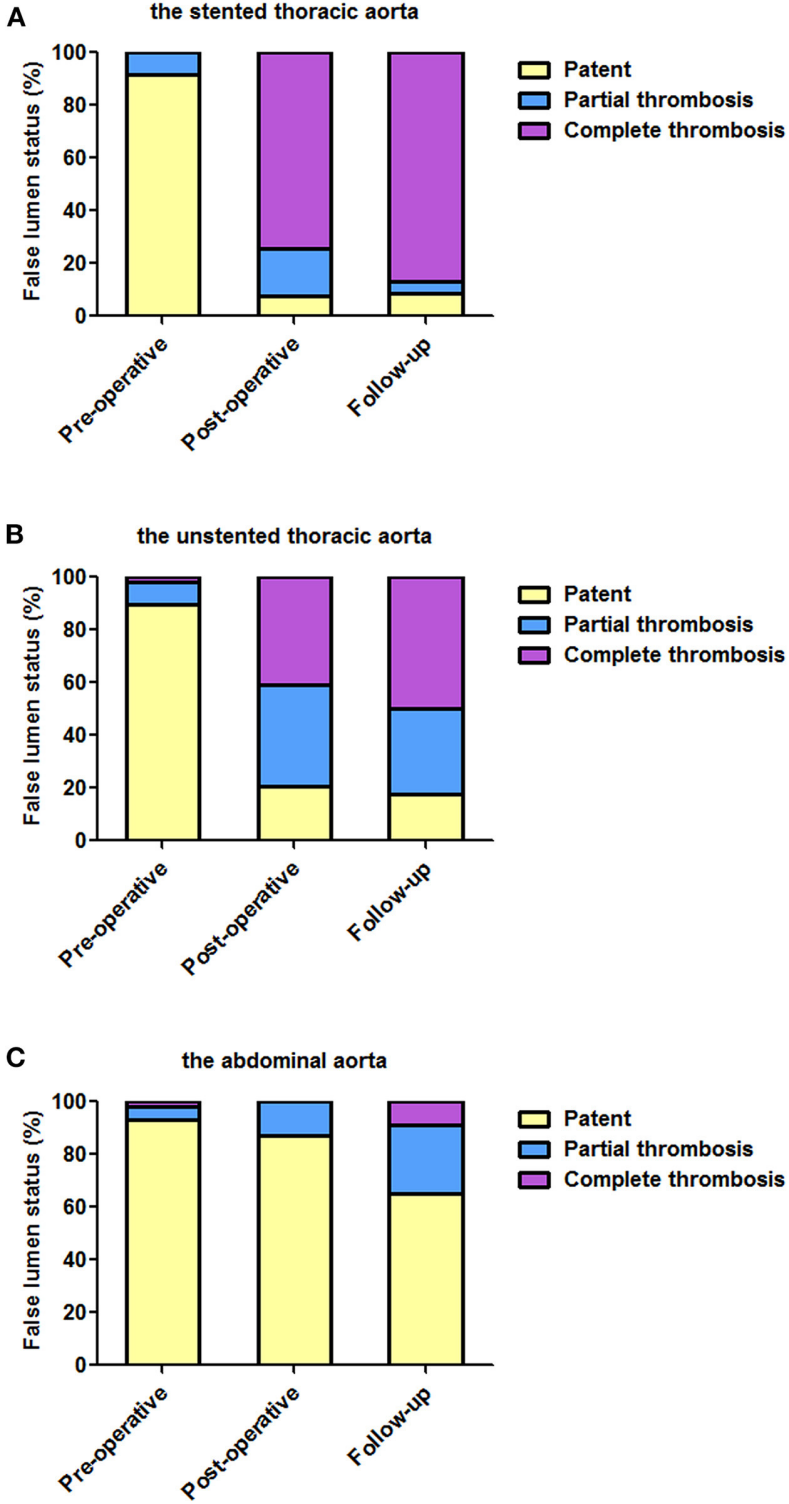
The status of FL over time at different aortic segments. **(A)** TEVAR induced the complete thrombosis of the stented thoracic FL in most of the patients. **(B)** After TEVAR, the unstented thoracic FL was completely thrombosed in approximately half of the patients before discharge and during follow-up. **(C)** FL in the abdominal aorta remained patent in most of the patients at the two post-TEVAR time-points. TEVAR, thoracic endovascular aortic repair; FL, false lumen; TL, true lumen.

Table 4 lists the FL status of three aortic segments pre-operatively before hospital discharge and during follow-up. The status of FL over time on the stented thoracic aorta (A), unstented thoracic aorta (B), and abdominal aorta (C) is shown in Figure 2.

**Table 4:** FL status in the stented thoracic aorta, unstented thoracic aorta, and abdominal aorta, which are categorized as patent, partial thrombosis, and complete thrombosis.

**Variables**	**Pre-operative**	**Post-operative**	**Follow-up**	***P-*value**
The stented thoracic aorta				<0.001
Patent	54 (91.5%)	3 (7.7%)	4 (8.7%)	
Partial thrombosis	5 (8.5%)	7 (17.9%)	2 (4.3%)	
Complete thrombosis (obliteration)	0 (0%)	29 (74.4%)	40 (87.0%)	
The unstented thoracic aorta				<0.001
Patent	53 (89.8%)	8 (20.5%)	8 (17.4%)	
Partial thrombosis	5 (8.5%)	15 (38.5%)	15 (32.6%)	
Complete thrombosis (obliteration)	1 (1.7%)	16 (41.0%)	23 (50.0%)	
The abdominal aorta				0.002
Patent	55 (93.2%)	34 (87.2%)	30 (65.2%)	
Partial thrombosis	3 (5.1%)	5 (12.8%)	12 (26.1%)	
Compete thrombosis (obliteration)	1 (1.7%)	0 (0.0%)	4 (8.7%)	

*All data are expressed as n (%)*.

### The Stented Thoracic Aorta

On pre-operative imaging, completely patent FL present in 91.5% of the patients. After TEVAR, partially thrombosed FL increased from 8.5% pre-operatively to 17.9% before discharge, and it was 4.3% during follow-up. Completely thrombosed FL persisted in 74.4 and 87.0% of the patients before discharge and during follow-up, respectively.

### The Unstented Thoracic Aorta

Completely patent FL significantly decreased from 89.8% at baseline to 20.5% before discharge and 17.4% during follow-up. Partially thrombosed FL increased from 8.5% at baseline to 38.5% before discharge, and it was 32.6% during follow-up. Totally, thrombosed FL substantially increased from 1.7% at baseline to 41% before discharge and to 50.0% during follow-up.

### The Abdominal Aorta

False lumen was maintained in 93.2, 87.2, and 65.2% of the patients pre-operatively, before discharge, and during follow-up, respectively. Partially thrombosed FL increased from 5.1% pre-operatively to 12.8% before discharge and then continued to increase to 26.1% during follow-up. Completely thrombosed FL was merely 8.7% during follow-up.

### Aortic Morphology: A Change in Lumen Diameters From Pre-operation

**Figure 3:**
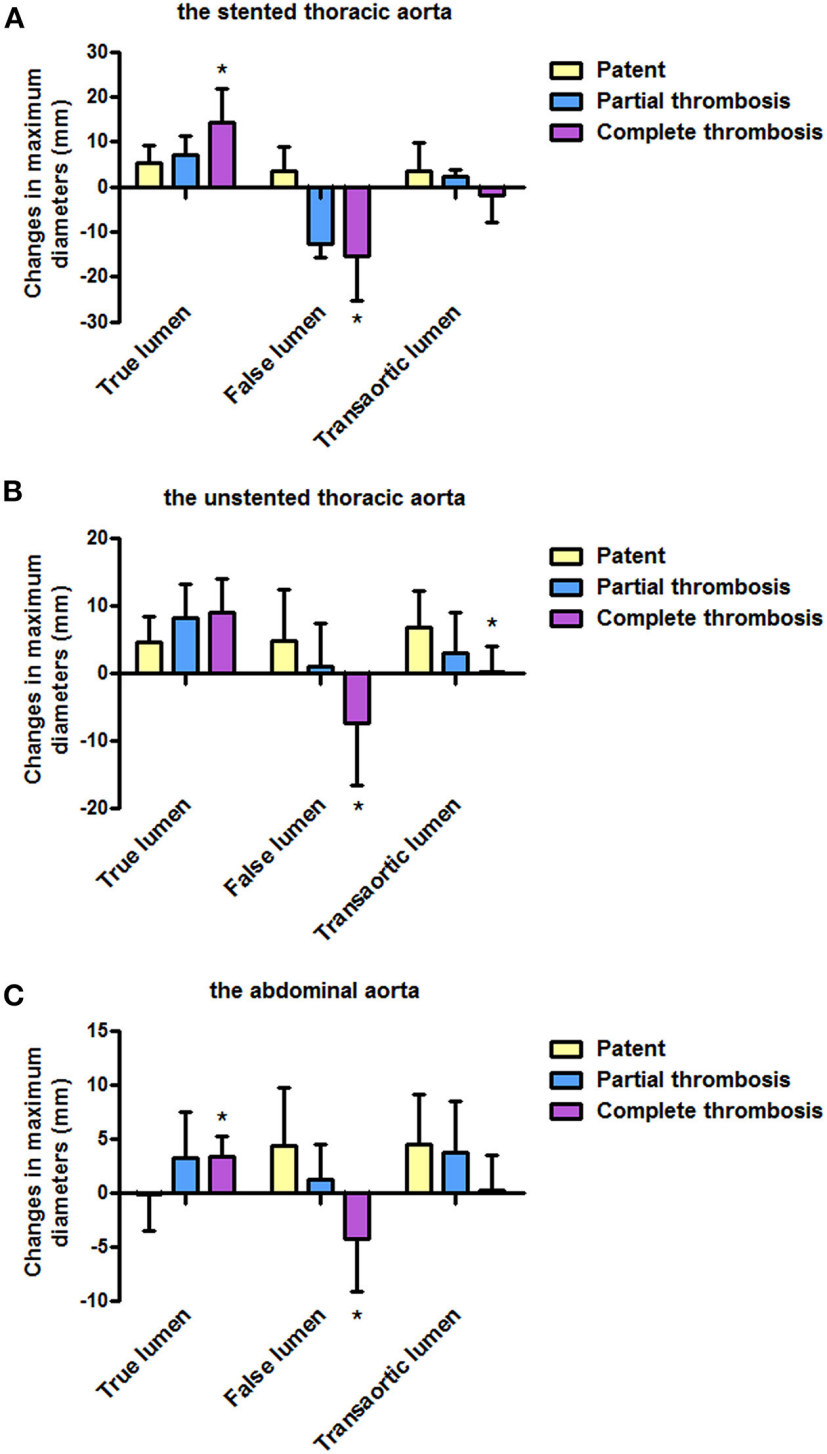
Changes in lumen diameters from a pre-operative measurement according to the FL status during follow-up. **(A)** Changes in TL and FL diameters on the stented thoracic aorta were more in patients with completely thrombosed FL than in patients with patent or partially thrombosed FL. **(B)** Compared with patients with patent or partially thrombosed FL, patients with completely thrombosed FL showed more obvious changes in FL and transaortic diameters on the unstented thoracic aorta. **(C)** Abdominal aorta over time showed more changes in TL and FL diameters in patients with completely thrombosed FL than in patients with patent or partially thrombosed FL. FL, false lumen; TL, true lumen. *P<0.05 versus partial thrombosis or patent.

According to FL status, patients who fully completed a long-term follow-up were divided into patent, partial thrombosis, and complete thrombosis. Table 5 shows changes in lumen diameters of TL, FL, and transaorta from a pre-operative measurement. Changes in lumen diameters on the stented thoracic aorta (A), unstented thoracic aorta (B), and abdominal aorta (C) between follow-up and pre-operation are shown in Figure 3.

**Table 5:** Changes in the maximum diameters of the TL, FL, and total lumen (transaortic) in the stented thoracic aorta, unstented thoracic aorta, and abdominal aorta according to FL status.

**Aortic Segment**	**Patent**	**Partial**	**Complete**	***P-*value**
		**thrombosis**	**thrombosis**	
The stented thoracic aorta				
True lumen	5.4 (3.9)	7.0 (4.4)	14.4 (7.5)	0.001
False lumen	3.6 (5.3)	−12.7 (3.1)	−15.3 (9.9)	0.002
Transaortic lumen	3.6 (6.1)	2.2 (1.5)	−2.0 (6.0)	0.156
The unstented thoracic aorta				
True lumen	4.5 (3.8)	8.2 (5.0)	8.9 (5.1)	0.100
False lumen	4.7 (7.6)	0.9 (6.4)	−7.4 (9.3)	0.001
Transaortic lumen	6.8 (5.4)	3.0 (5.9)	0.2 (3.8)	0.006
The abdominal aorta				
True lumen	−0.2 (3.4)	3.2 (4.2)	3.3 (1.9)	0.012
False lumen	4.3 (5.4)	1.2 (3.2)	−4.3 (4.9)	0.004
Transaortic lumen	4.5 (4.6)	3.7 (4.8)	0.2 (3.2)	0.217

*All data are expressed as means (SD) in mm*.

### The Stented Thoracic Aorta

Average increases in TL diameters were 7.0 and 14.4 mm in patients with partially and completely thrombosed FL, respectively, and exceed the value of 5.4 mm in patients with patent FL (*p* = 0.001). Average FL diameters decreased by 12.7 and 15.3 mm in patients with partially and completely thrombosed FL, respectively, whereas they are raised by 0.6 mm in patients with patent FL (*p* = 0.002). Average change in transaortic diameters did not statistically differ among patients with patent, partially thrombosed, and completely thrombosed FL (*p* = 0.156).

### The Unstented Thoracic Aorta

Average increases in TL diameters were 4.5, 8.2, and 8.9 mm in patients with patent, partially thrombosed, and completely thrombosed FL, respectively, although there was no statistical significance (*p* = 0.100). Average FL diameter was decreased by 7.4 mm in patients with completely thrombosed FL, whereas it was increased by 0.9 and 4.7 mm in patients with patent FL and partially thrombosed FL (*p* = 0.001). Average increase in transaortic diameters was 0.2 mm in patients with completely thrombosed FL, which was <3.0 mm in patients with partially thrombosed FL and 6.8 mm in patients with patent FL (*p* = 0.006).

### The Abdominal Aorta

Average TL diameters were increased by 3.3 and 3.2 mm in patients with partially and completely thrombosed FL, respectively, whereas they were decreased by 0.2 mm in patients with patent FL (*p* = 0.012). Average FL diameter was decreased by 4.3 mm in patients with completely thrombosed FL, whereas it was increased by 1.2 mm and 4.3 in patients with partially thrombosed and patent FL, respectively (*p* = 0.004). There were no statistical differences on the average increase in transaortic diameters among patients with patent, partially thrombosed, or completely thrombosed FL (*p* = 0.217).

### Aortic Remodeling: Representative CTA During a Long-Term Follow-Up

**Figure 4:**
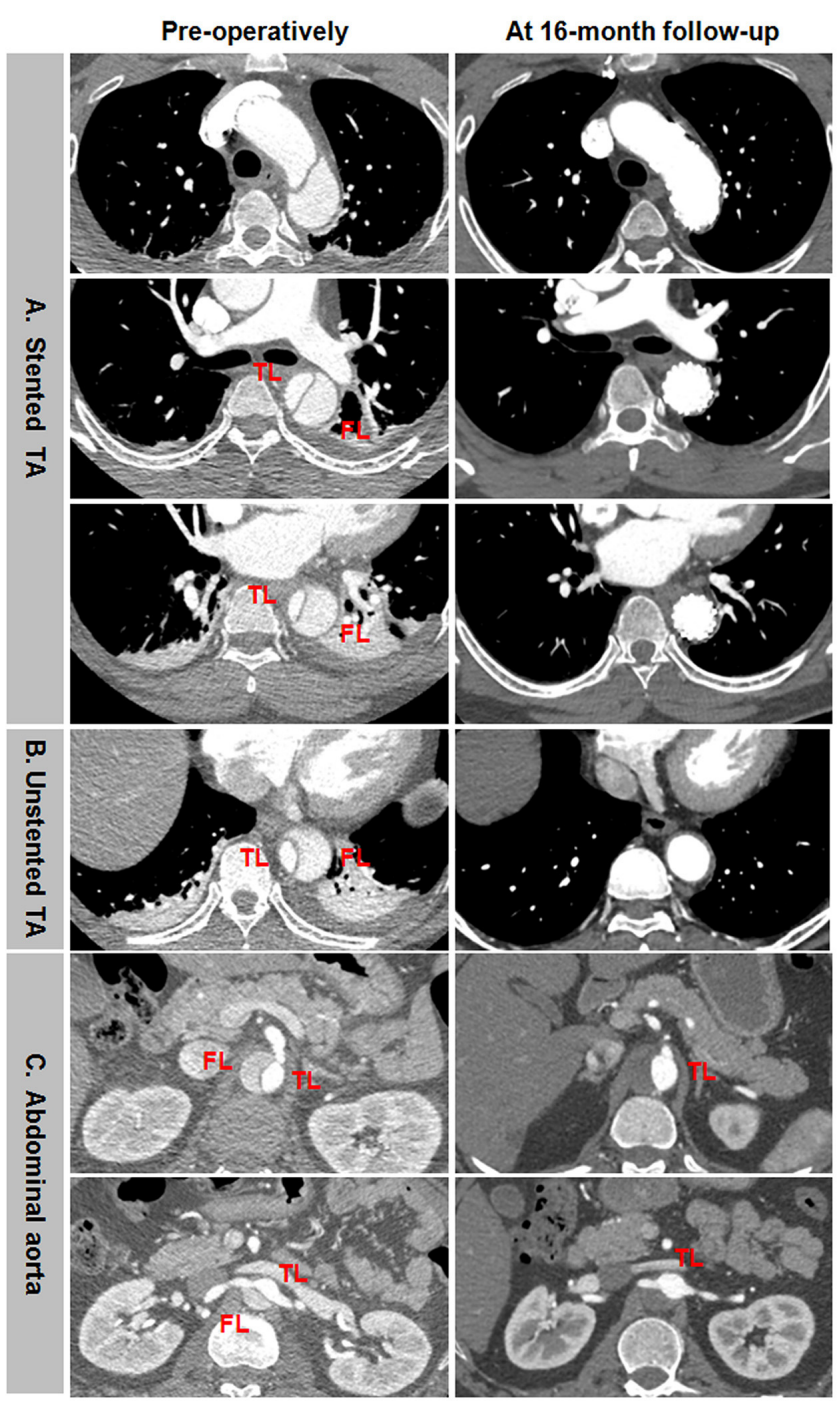
Favorable aortic remodeling after TEVAR for TBAD in the thoracic and abdominal aorta. **(A,B)** FL was completely thrombosed and regressed, and TL was enlarged on the stented and unstented thoracic aorta in 13 months after TEVAR. **(C)** The entire thrombosis of FL and the stabilization of TL occurred on the abdominal aorta during a 13-month follow-up. TBAD, type B aortic dissection; TEVAR, thoracic endovascular aortic repair; FL, false lumen; TL, true lumen.

Favorable aortic remodeling included FL thrombosis, FL obliteration, TL enlargement, and transaortic stabilization (Figure 4). In a 13-month follow-up, FL was entirely thrombosed and regressed, and TL was totally recovered over the whole stented and unstented thoracic aorta (Figures 4A,B). FL was completely thrombosed but did not shrink, and TL was relatively stabilized over the abdominal aorta in 13 months after TEVAR (Figure 4C).

**Figure 5:**
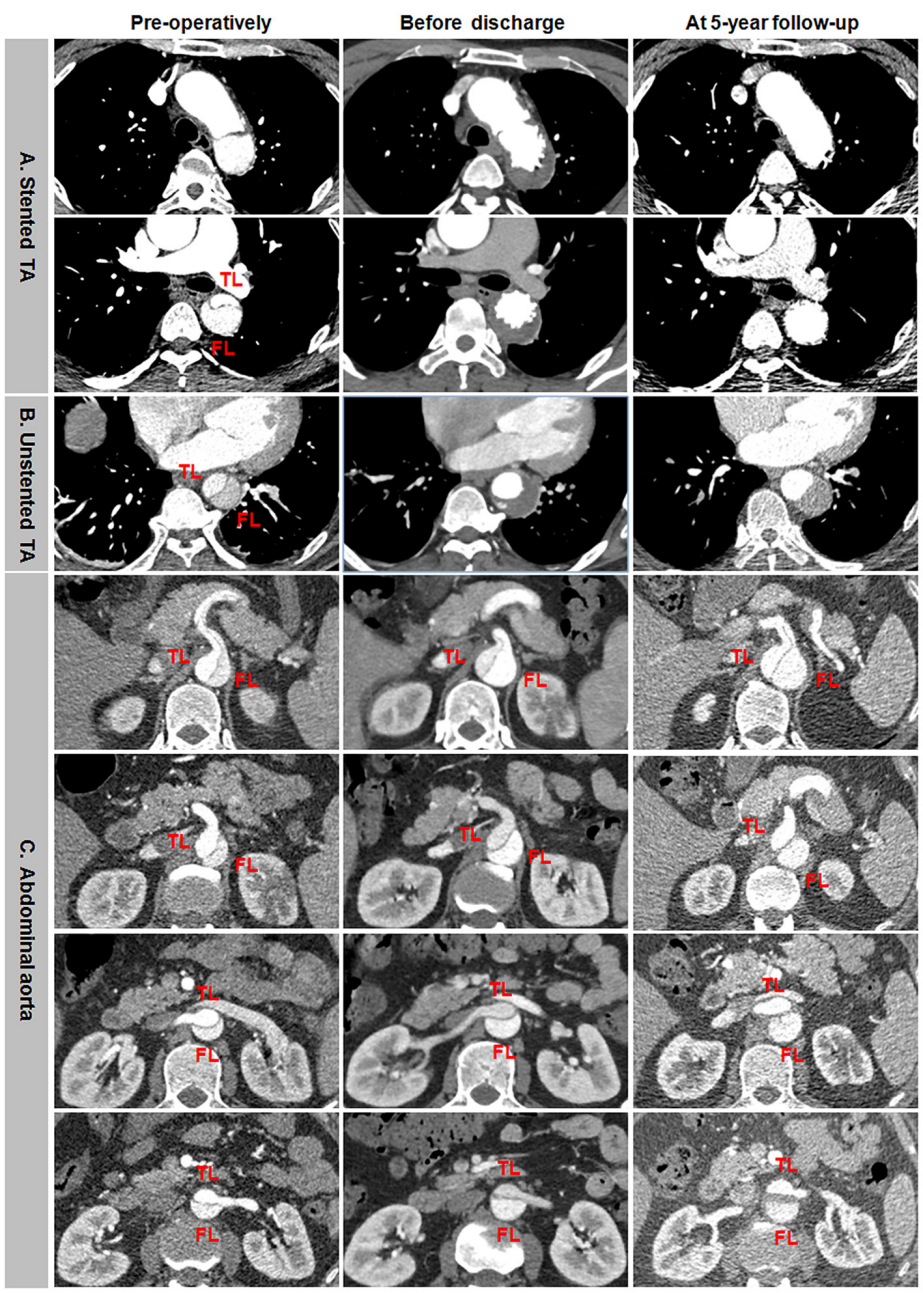
Differential remodeling patterns between the thoracic and abdominal aorta after TEVAR for TBAD. **(A)** FL was entirely thrombosed immediately after TEVAR, and the absorption of FL thrombosis and the enlargement of TL occurred on the stented thoracic aorta in 5 years post-operatively. **(B)** FL was thrombosed immediately after TEVAR, and the progressive dilation of thrombosed FL was on the unstented thoracic aorta in 5 years after TEVAR. **(C)** FL remained patent, and TL was relatively stabilized immediately after TEVAR, an obvious enlargement of patency FL and the resultant transaortic expansion occurred on the abdominal aorta in 5 years after TEVAR. TBAD, type B aortic dissection; TEVAR, thoracic endovascular aortic repair; FL, false lumen; TL, true lumen.

Incomplete aortic remodeling was defined as FL patency and expansion, TL stabilization, and transaortic growth at the downstream abdominal aorta (Figure 5). Over the stented thoracic aorta, FL was entirely thrombosed immediately after TEVAR, and FL was obliterated and TL was completely recovered due to the absorption of thrombose in a 5-year follow-up (Figure 5A). FL thrombosis and TL expansion on the unstented thoracic aorta occurred immediately and in a 5-year post-operation (Figure 5B). A progressive growth in the abdominal aorta after TEVAR was mainly attributed to an expansion of FL, whereas TL remained unchanged in size (Figure 5C).

**Figure 6:**
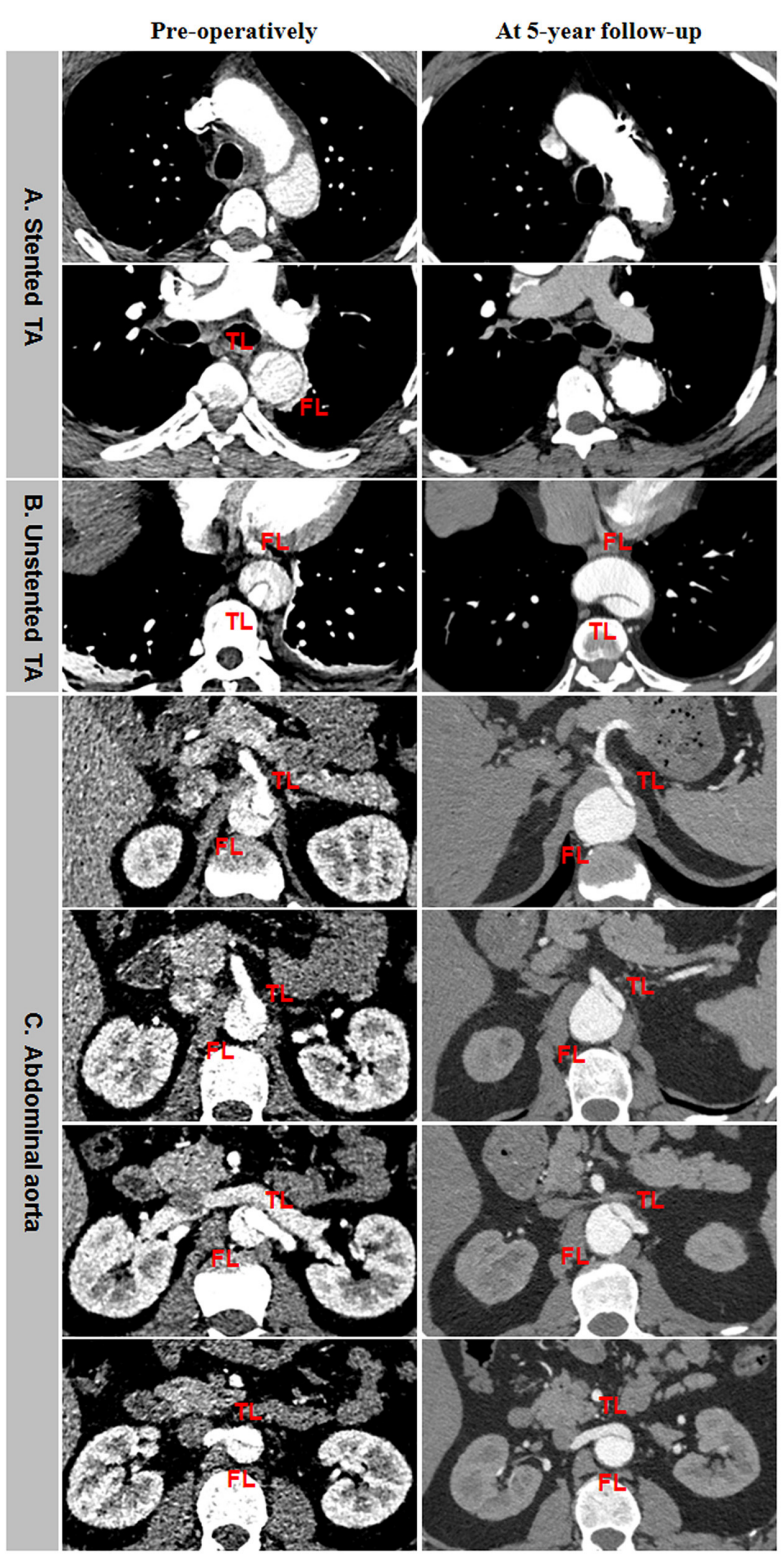
Abdominal aneurismal expansion during a long-term follow-up after TEVAR for TBAD. **(A)** FL was completely thrombosed and obliterated on the stented thoracic aorta during a 5-year follow-up. **(B)** The persistent patency and obvious enlargement of FL occurred on the unstented thoracic aorta in a 5-years post-operation. **(C)** Aneurysmal degeneration of the distal abdominal aorta was attributed to TL shrinkage and FL expansion in 5 years after TEVAR. TBAD, type B aortic dissection; TEVAR, thoracic endovascular aortic repair; FL, false lumen; TL, true lumen.

Aneurysmal dilation of aorta distal to the stent graft was attributed to persistent FL patency, pressurization, enlargement, and TL shrinkage (Figure 6). Total thrombosis and obliteration of the FL were seen in the stented thoracic aorta in a 5-year follow-up (Figure 6A). FL in the unstented thoracic aorta remained patent and was obviously enlarged in a 5-year follow-up (Figure 6B). Aneurysmal expansion of the downstream abdominal aorta was a combination of TL shrinkage and FL expansion in 5 years after TEVAR (Figure 6C).

### Abdominal Aortic Branch Remodeling

**Figure 7:**
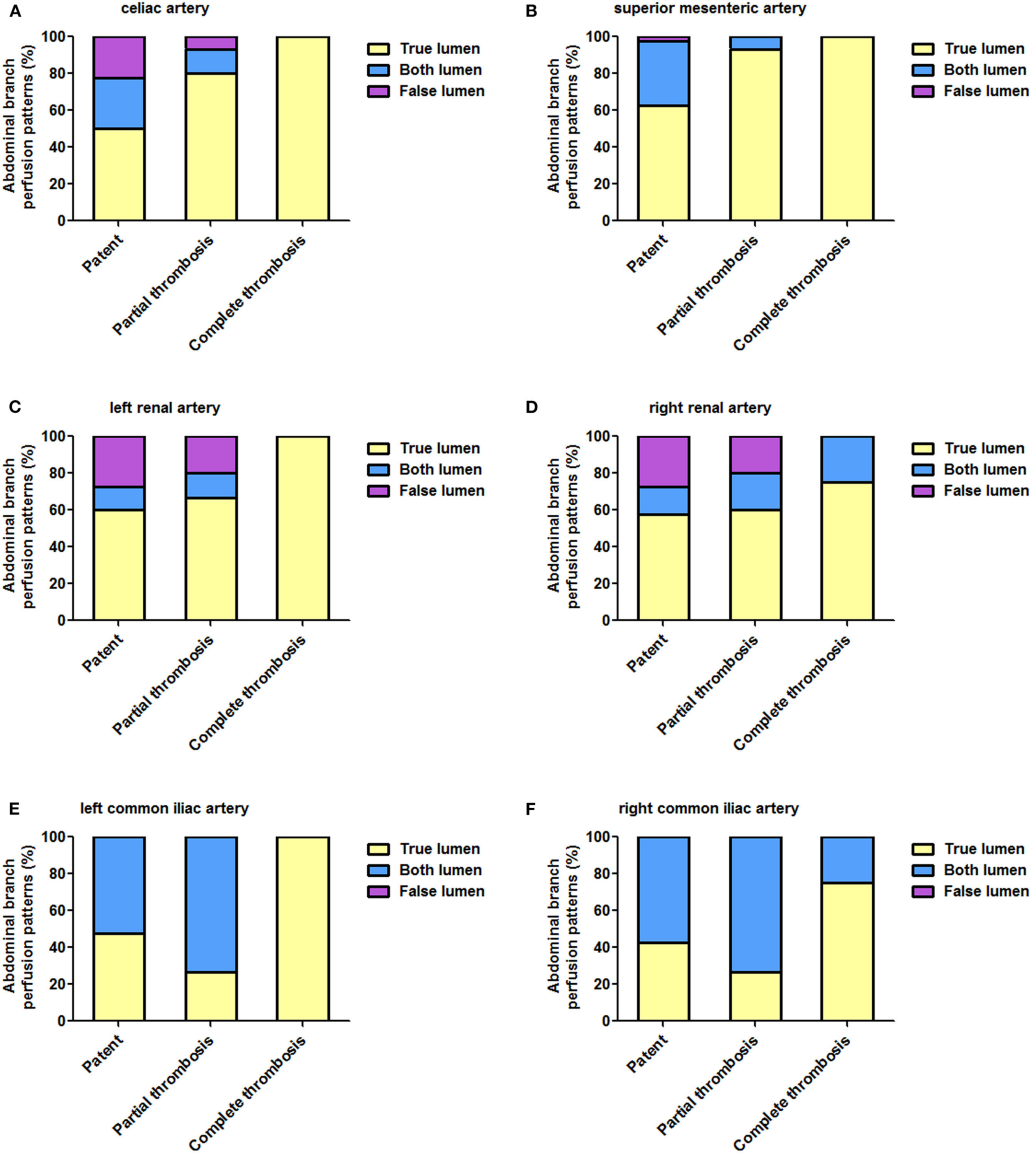
Abdominal aortic branch perfusion patterns according to FL status during follow-up. **(A–E)** The percentage of TL perfusion on all abdominal branches was higher in patients with completely thrombosed FL than in patients with patent or partially thrombosed FL, and this difference reached a statistical significance on celiac **(A)**, superior mesenteric **(B)**, and left common iliac **(E)** arteries. FL, false lumen; TL, true lumen.

Fifty-nine patients provided 354 branches for a review and an analysis. Table 6 list abdominal aortic branch perfusion patterns pre-operatively and during a more recent follow-up. Perfusion patterns of celiac (A), superior mesenteric (B), left renal (C), right renal (D), left common iliac €, and right common iliac arteries according to FL status during follow-up are shown in Figure 7.

**Table 6:** Abdominal branch perfusion patterns in the celiac artery, superior mesenteric artery (SMA), left renal artery (LRA), right renal artery (RRA), left common iliac artery, and right common iliac artery according to FL status.

**Abdominal branches**	**Pre-operative**	**Follow-up**	**Follow-up**			
			**Patent**	**Partial thrombosis**	**Complete thrombosis**	***P-*value**
Celiac artery						
True lumen	31 (52.5%)	36 (61.0%)	20 (50.0%)	12 (80.0%)	4 (100%)	0.041
Both lumens	17 (28.8%)	13 (22.0%)	11 (27.5%)	2 (13.3%)	0 (0.0%)	0.498
False lumen	11 (18.6%)	10 (17.0%)	9 (22.5%)	1 (6.7%)	0 (0.0%)	0.344
Superior mesenteric artery						
True lumen	39 (66.1%)	43 (72.9%)	25 (62.5%)	14 (93.3)	4 (100%)	0.033
Both lumens	18 (30.5%)	15 (25.4%)	14 (35.0%)	1 (6.7%)	0 (0.0%)	0.040
False lumen	2 (3.4%)	1 (1.7%)	1 (2.5%)	0 (0.0%)	0 (0.0%)	0.685
Left renal artery						
True lumen	37 (62.7%)	38 (64.4%)	24 (60.0%)	10 (66.7%)	4 (100%)	0.398
Both lumens	11 (18.6%)	7 (11.9%)	5 (12.5%)	2 (13.3%)	0 (0.0%)	1.000
False lumen	11(18.6%)	14 (23.7%)	11 (27.5%)	3 (20.0%)	0 (0.0%)	0.685
Right renal artery						
True lumen	32 (54.2%)	35 (59.3%)	23 (57.5%)	9 (60.0%)	3 (75.0%)	0.913
Both lumens	13 (22.0%)	10 (17.0%)	6 (15.0%)	3 (20.0%)	1 (25.0%)	0.612
False lumen	14 (23.7%)	14 (23.7%)	11 (27.5%)	3 (20.0%)	0 (0.0%)	0.685
Left common iliac artery						
True lumen	26 (44.1%)	27 (45.8%)	19 (47.5%)	4 (26.7%)	4 (100%)	0.024
Both lumens	33 (55.9%)	32 (54.2%)	21 (52.5%)	11 (73.3%)	0 (0.0%)	0.024
False lumen	0 (0.0%)	0 (0.0%)	0 (0.0%)	0 (0.0%)	0 (0.0%)	NA
Right common iliac artery						
True lumen	29 (49.2%)	24 (40.7%)	17 (42.5%)	4 (26.7%)	3 (75.0%)	0.229
Both lumens	30 (50.8%)	35 (59.3%)	23 (57.5%)	11(73.3%)	1 (25.0%)	0.229
False lumen	0 (0.0%)	0 (0.0%)	0 (0.0%)	0 (0.0%)	0 (0.0-)	NA

*All data are expressed as n (%)*.

The baseline branch perfusion pattern was given as follows: 54.8% (194/354) TL branches, 34.5% (122/354) TL and FL branches, and 10.8% (38/354) FL branches. Among the abdominal aortic branches, right renal arteries were most frequently involved with dissection (23.7% by FL and 22.0% by TL and FL), whereas SMA was most often perfused entirely by a TL (66.1%).

Branch remodeling after TEVAR was characterized as positive in 22 branches: 8 celiac, 6 SMA, 2 left renal, 5 right renal, and 1 left common iliac arteries. Around 4.5% (16/354) of branch supplies switched from BL into TL, 0.8% (3/354) of the branch perfusion was in transit from FL to TL, and 0.8% (3/354) of the branch perfusion changed from FL to BL. Negative branch remodeling was seen in 15 branches: 3 celiac, 3 left renal, 3 right renal, 1 left common iliac, 5 right common iliac arteries. Around 3.1% (11/354) of the branch perfusion switched from BL to FL, 0.8% (3/354) of the perfusion pattern changed from TL to BL, and 0.3% (1/354) of branches altered from TL to BL.

According to FL status, patients were classified as patent, partial thrombosis, and complete thrombosis. Around 50 and 80% of celiac arteries originated from TL in patients with patent and partially thrombosed FL, respectively, but the same was perfused entirely by TL in patients with completely thrombosed FL (*p* = 0.041). Comparing with 62.5% TL support in patients with patent FL, SMA was perfused by TL in 93.3 and 100% of patients with partially and completely thrombosed FL (*p* = 0.033). Left renal arteries were perfused by TL in 60.0, 66.7, and 100% of patients with patent, partially thrombosed, or completely thrombosed FL, respectively (*p* = 0.398). Right renal arteries were perfused by TL in 57.5, 60.0, and 75.0% of patients with patent, partially thrombosed, or completely thrombosed FL, respectively (*p* = 0.913). Around 47.5, 26.7, and 100% of left common iliac arteries started from TL in patients with patent, partially thrombosed, and completely thrombosed FL, respectively (*p* = 0.024). Around 42.5, 26.7, and 75% of right common iliac arteries started from TL in patients with patent, partially thrombosed, and completely thrombosed FL, respectively (*p* = 0.229).

**Figure 8:**
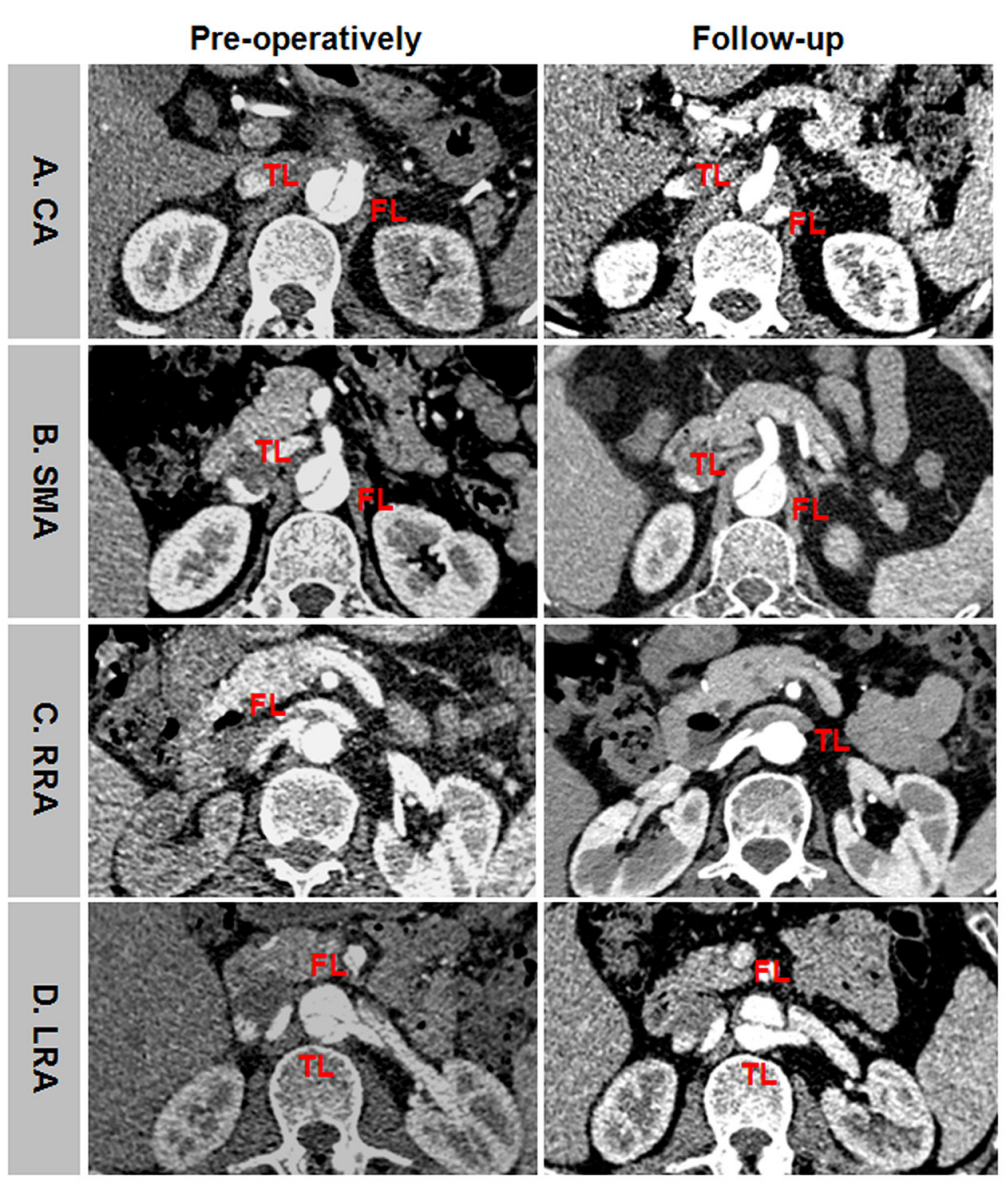
An example of positive branch remodeling after TEVAR. **(A)** CA was perfused by FL at baseline and by TL during follow-up. **(B)** SMA was perfused by BL at baseline and by TL during follow-up. **(C)** RRA was supported by FL at baseline and by TL during follow-up. **(D)** LRA was supported by BL at baseline and by TL during follow-up. TEVAR, thoracic endovascular aortic repair; FL, false lumen; TL, true lumen; BL, bilateral lumen; CA, celiac artery; SMA, superior mesenteric artery; RRA, right renal artery; LRA, left renal artery.

**Figure 9:**
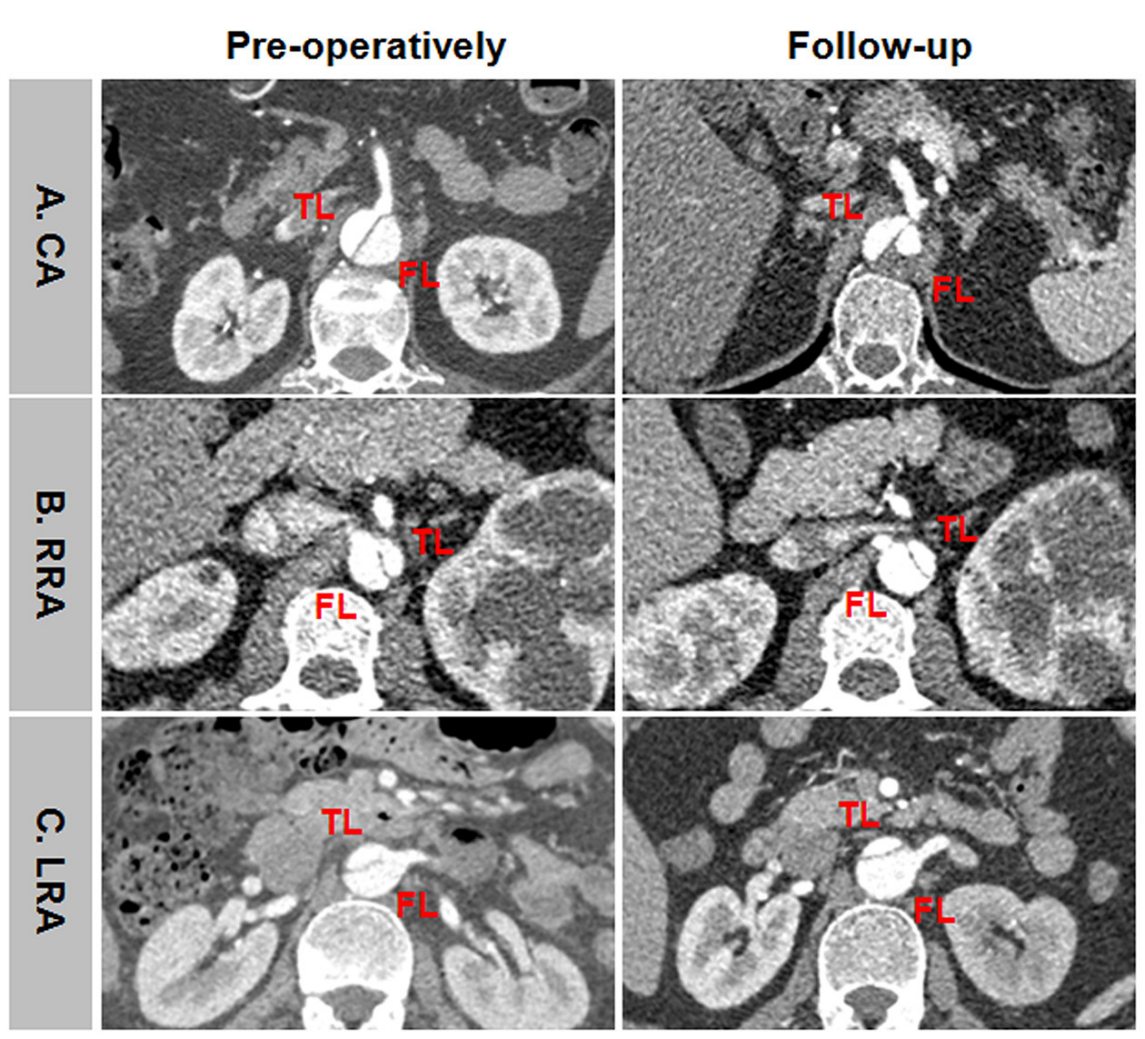
An example of negative branch remodeling after TEVAR. **(A)** CA was supplied by TL at baseline and by BL during follow-up. **(B)** RRA was perfused by BL at baseline and by FL during follow-up. **(C)** LRA was supported by BL at baseline and by FL during follow-up. TEVAR, thoracic endovascular aortic repair; FL, false lumen; TL, true lumen; BL, bilateral lumen; CA, celiac artery; RRA, right renal artery; LRA, left renal artery.

Representative positive branch remodeling is shown in Figure 8. Celiac arterial supply changed from FL to TL after TEVAR (Figure 8A). SMA perfusion pattern altered from BL to TL after TEVAR (Figure 8B). Right renal artery (RRA) was in transit from FL at baseline and TL during follow-up (Figure 8C). Left renal artery (LRA) switched from BL at baseline to TL during follow-up (Figure 8D). Representative negative branch remodeling is indicated in Figure 9. Celiac arterial supply changed from TL to BL after TEAVR (Figure 9A). RRA switched from BL into FL after TEVAR (Figure 9B). LRA was in transit from BL into FL after TEVAR (Figure 9C).

## Discussion

Medical management alone for acute TBAD had a long-term consequence of aneurismal degeneration and aortic rupture in up to 40% of the patients (9). This is prevented by TEVAR-induced aortic remodeling around the stented segment of aorta (10). However, less attention has been paid to structural changes in the thoracic and abdominal aorta below the stent, and only a few studies have been involved (11–14). Our study indicated that the maximum diameter of the stented thoracic aorta is stable with an increase of the diameter of stented TL and a decrease the diameter of stented FL, increased maximal diameters of the unstented thoracic aorta are secondary to TL augmentation, and the aneurysmal growth of the unstented abdominal aorta was mainly attributed to FL expansion. Wojciechowski et al. indicated that total aortic diameter and area are stable in the stent-covered thoracic aorta but significantly increase at the aorta distal to the stent due to TL augmentation in an average follow-up of 57.9 months (11). Conrad et al. reported that TEVAR stabilized the maximal aortic diameter of the stented segment, and the growth of the unstented thoracic aorta was mainly attributed to TL expansion (12). Lombardi et al. observed a transaortic growth >5 mm for abdominal aorta in 33.3 and 46.7% of the patients in 12 and 24 months post-operatively, mostly attributed to an expansion in FL (13). Leshnower et al. exhibited no significant changes in the diameter of the stented thoracic aorta after TEVAR, and that increase in aortic diameters observed in the downstream abdominal segments was a combination of TL and FL expansion distal to the stent (14). Persistent robust flow through a distal entry tear maintains the continued FL pressurization, and may lead to its expansion. TL expansion responds to a change in hemodynamics after TEVAR. Based on these findings, long-term aortic dilation, predominantly in the abdominal aorta, is a phenomenon that must be closely observed on follow-up imaging.

Thoracic endovascular aortic repair in acute TBAD covers the primary entry tear and initiates FL thrombosis and TL expansion. Complete FL thrombosis excludes FL from the circulation and is thought to be a principle on which TEVAR is based, whereas patients with a patency or partial thrombosis of FL have an increased risk of aortic expansion and death (15–17). In this study, complete FL thrombosis during follow-up was observed in 87, 50, and 8.7% of the patients in the stented thoracic aorta, unstented thoracic aorta, and abdominal aorta, respectively. From baseline to follow-up, FL diameter for the dissected aorta was substantially reduced specially in patients with complete FL thrombosis, whereas it was enlarged in patients with patent FL. The increase in TL diameters was significantly larger in patients with partial and complete FL thrombosis than in patients with patent FL, with a statistical difference seen during follow-up for the abdominal aorta. Conrad et al. reported that FL thrombosis had a significant effect on the reduction of aortic growth as aortic growth of the abdominal aorta was 31% in patients with a patent FL vs. 3% in those with a thrombosed FL (12). Lombardi et al. indicated that all thoracic FLs are partially or completely thrombosed after a 30-day follow-up while the rates of complete thrombosis in the abdominal FL are lower (13). Leshnower et al. indicated that complete FL thrombosis and obliteration are 100% in the stented thoracic aorta and 78% in the unstented thoracic aorta in 12 months after TEVAR, and abdominal aortic FL remained patent in 78% of the patients (14). A systemic review involving 16 trials indicated that total thrombosis of FL is seen in 80.6–90% of the patients at the level of stent graft, and below the level of the diaphragm total thrombosis of the FL is less frequent (22–76.5%) (2). Sigman et al. reported that patients with FL thrombosis achieved abdominal TL expansion and FL regression after TEVAR with no changes in the abdominal aortic volume, and abdominal aortic volume significantly increased in patients with FL patency (18). Total obliteration of FL appears to be a key factor to aneurismal prevention as aortic growth in the unstented aorta mainly occurs in patients with a patent FL. A failure to achieve thoracic FL thrombosis negatively influences aortic morphological outcome of the abdominal aorta after TEVAR for acute TBAD.

More attention has been paid to identify the factors that prevent the development of complete thrombosis in FL after TEVAR in patients with TBAD. Clarifying the factors that could affect the thrombus formation at FL has major importance for predicting the long-term outcome of patients with extensive TBAD undergoing TEVAR. The number of visceral branches that originated partially or totally from the FL was a significant independent risk factor associated with incomplete thrombosis in FL after TEVAR (19–21). Retrograde perfusion of FL can be established by perfusion through the involvement of branch vessels. Multiple tears were located in the abdominal aorta, which allowed continued perfusion of FL after TEVAR, was an independent risk factor for incomplete FL thrombosis after TEVAR (19–21). Decreased pre-operative TL area ratio in the abdominal aorta is a predictive factor of patent FL after TEVAR (22). The bigger pre-operative TL in the abdominal aorta, the better the aortic remodeling. The smaller pre-operative abdominal TL is also compatible with a higher continuous FL pressure as a result of blood flow from multiple reentries and branch vessels at the level of the abdominal aorta.

This study has demonstrated that the natural course of the abdominal braches after TEVAR is largely benign. After TEVAR, 89.5% (317/354) of the branches remained unchanged in their perfusion patterns. Of the remaining 37 branches, 22 branches (6.2%; 22/354) demonstrated an increased TL perfusion, and 15 branches (4.2%; 15/354) had an increased FL contribution. This is in agreement with a clinical investigation of Han et al. (22) demonstrating a review of 46 patients who underwent TEVAR for TBAD and analyzing 295 abdominal aortic branches, changes in perfusion patterns were observed in 16 (5.4%; 16/295) patients, 12 branches (4.1%; 12/295) had an increased TL contribution to perfusion, 4 branches (1.4%; 4/295) had an increased FL contribution. In a recent study, two renal artery branch interventions have been occurred based on the radiographic appearance of the branch perfusion. This low rate of branch interventions (0.5%; 2/354) is consistent with the report by Magee et al. (23) who demonstrated that the branches that were patent before TEVAR almost always remain patent after TEVAR, and <5% of branch vessels required reintervention. Around 91.5% of the patients had a patent FL in the abdominal aorta over a long follow-up period. Persistent robust filling of FL through multiple reentries in the abdominal aorta is the main reason for unchanged abdominal aortic branch perfusion patterns because TEVAR has occluded a proximal entry tear.

## Limitations

There are several limitations to this study. Small numbers of patients and the lack of a longer follow-up unfavorably affect the results of the remodeling analysis. It remains a persistent challenge to maintain CT follow-up in patients, particularly when they become asymptomatic. This study did not investigate whether bare-metal stent or FL embolization improves the efficacy of TEVAR. The data from this study add to a growing body of published reports evaluating the aortic remodeling and abdominal aortic branch perfusion patterns after TEVAR-TBAD. Complete FL thrombosis might be misjudged and exaggerated when an contrast agent did not fill and enhance FL despite the absence of a real intraluminal thrombus.

## Conclusion

Successful TEVAR provides favorable aortic remodeling in the stent-covered thoracic aorta, which are manifested as TL enlargement, FL thrombosis and regression, and transaortic stabilization. Distal to the stent graft, the abdominal FL remains patent; increases in aortic diameters observed in the abdominal aorta were secondary to FL expansion. After TEVAR, abdominal branch perfusion remains largely stable with a less intervention rate. Abdominal aneurysmal dilation and an unchanged branch perfusion pattern are mainly attributed to continuous FL patency, pressurization, and augmentation as a result of retrograde perfusion from the remaining intimal tears.

## Data Availability Statement

The raw data supporting the conclusions of this article will be made available by the authors, without undue reservation.

## Ethics Statement

The studies involving human participants were reviewed and approved by the Institutional Review Board of Union Hospital, Tongji Medical College, Huazhong University of Science and Technology, Wuhan, China. Written informed consent for participation was not required for this study in accordance with the national legislation and the institutional requirements. Written informed consent was not obtained from the individual(s) for the publication of any potentially identifiable images or data included in this article.

## Author Contributions

ZY: collection and assembly of data, data analysis, and interpretation. YL: administrative support, conception and design, and final approval of the manuscript. BJ: provision of the study material or patients, conception, and design. JW: financial support, conception and design, manuscript writing, and final approval of the manuscript. All authors contributed to the article and approved the submitted version.

## Funding

This work was supported by the National Natural Sciences Foundation of China [Grant No. 81770277].

## Conflict of Interest

The authors declare that the research was conducted in the absence of any commercial or financial relationships that could be construed as a potential conflict of interest.

## Publisher's Note

All claims expressed in this article are solely those of the authors and do not necessarily represent those of their affiliated organizations, or those of the publisher, the editors and the reviewers. Any product that may be evaluated in this article, or claim that may be made by its manufacturer, is not guaranteed or endorsed by the publisher.
